# Bifid mandibular condyle: investigating the association with temporomandibular joint disorders on three-dimensional T2-weighted SPACE sequences performed for brain magnetic resonance radiological scanning techniques

**DOI:** 10.22514/jofph.2026.022

**Published:** 2026-03-12

**Authors:** Rumeysa Samanci, Hayri Ogul, Sarfinaz Ataoglu

**Affiliations:** ^1^Department of Physical Medicine and Rehabilitation, Medical Faculty, Duzce University, 81620 Duzce, Turkey; ^2^Department of Radiology, Medical Faculty, Istanbul Medipol University, 34810 Istanbul, Turkey

**Keywords:** Bifid mandibular condyle, Temporomandibular joint, T2-weighted SPACE sequence

## Abstract

**Background**: Bifid mandibular condyle (BMC) is a rare anatomical variation whose cause has not been fully understood. It has been hypothesized that BMC may be associated with an increased frequency of temporomandibular joint (TMJ) abnormalities. The aim of the study was to comparatively review the magnetic resonance imaging (MRI) archives of the hospital focusing on patients with BMC who underwent brain MRI using the T2-weighted 3D sampling perfection with application-optimised contrast using different flip angle evolutions (SPACE) radiological scanning techniques. **Methods**: The TMJ was thoroughly evaluated in patients diagnosed with BMC. Pathological findings were categorized into six classes: disc degeneration, disc displacement, disc deformation, degenerative osteoarthritis, effusion, and subluxation. **Results**: A total of 29 patients with BMC were identified, including 9 males (31%) and 20 females (69%). BMC was located on the right side in 10 patients (34.5%), on the left side in 7 patients (24.1%), and bilaterally in 12 patients (41.4%). Only 5 patients (17.2%) showed no pathological findings on MRI, while 24 patients (82.8%) exhibited at least one TMJ-related abnormality. Among these, 22 patients (75.9%) had disc degeneration, 8 (27.6%) had disc displacement, 10 (34.5%) had disc deformation, 2 (6.9%) had degenerative osteoarthritis, 12 (41.4%) had effusion, and 5 (17.2%) had subluxation. **Conclusions**: TMJ disc pathology is the most frequently observed abnormality in BMC, although it remains uncertain whether this relationship is causal or coincidental. BMC may contribute to the development of TMJ disorders; however, further research with larger sample sizes is needed to clarify its role.

## 1. Introduction

The mandible is the only movable bone in the skull and consists of two main components: the mandibular body and the mandibular ramus [1, 2]. The mandibular ramus features the coronoid and condylar processes. The condylar process functions as an articular element of the mandible and forms the temporomandibular joint (TMJ) by articulating with the mandibular fossa of the temporal bone [2, 3, 4]. Any malformation or variation in the bony or articular components of the TMJ can alter the function of this region [1, 5]. A rare variant is the bifid mandibular condyle (BMC), in which the mandibular condyle head is divided into two articular heads by a cleft or groove [6, 7]. The first documented case of BMC in a living individual was reported by Schier in 1948 [8, 9]. Since then, a growing number of cases have been described, primarily identified incidentally during routine orthopantomogram (OPG) radiographic examinations for dental evaluations [10, 11].

The BMC is an extremely rare condition, and its cause has not been fully clarified. It is believed to be either developmental or caused by trauma [12, 13]. Although advances in imaging techniques have led to an increase in detection rates of BMC, ranging from approximately 0.31% to 1.82%, the reported prevalence of BMC varies across studies, and BMC remains a rare condition [1, 14, 15]. Due to its generally asymptomatic nature, BMC has predominantly been documented in the literature through case reports [6, 16].

In some patients, identification of bifid condyles via OPG may be limited due to the two poles of the BMC not being visible and the inability of OPG to fully evaluate the condylar anatomy [10, 17]. Furthermore, panoramic radiographs may overestimate or underestimate bifidity due to overlapping anatomical structures or image distortion [1, 18]. Consequently, BMCs are often detected incidentally during radiographic examinations, and their identification has become more commonplace with the widespread use of advanced radiological imaging techniques [7, 10, 19]. Magnetic resonance imaging (MRI) and computed tomography (CT) are the most effective radiological methods for detecting bone mineral content (BMC) [13]. A three-dimensional (3D) assessment of BMCs enhances the reliability of such evaluations further [5, 17]. However, unlike MRI, CT does not allow visualization of the disc or other soft tissues and exposes patients to ionizing radiation. Consequently, BMCs are more likely to be discovered incidentally during an MRI examination of the head and neck region [12].

Three-dimensional sampling perfection with application-optimised contrasts using different flip-angle evolution (3D SPACE) sequences, which were first described by Mugler and his colleagues, have become increasingly frequent in routine practice, thanks to their excellent spatial and contrast resolution [20, 21, 22].

The detection of BMC in asymptomatic individuals is significant from a clinical perspective. Although this anatomical variation is often identified incidentally during diagnostic imaging scans, changes to the morphology of the condyle may affect TMJ function and increase the risk of joint sounds, pain, or restricted mandibular movement. Therefore, identifying BMC even in patients without symptoms may facilitate the early identification of those at risk of TMJ pathology and provide valuable guidance for clinical monitoring. In this study, brain MRI examinations acquired using the T2-weighted 3D SPACE sequence were retrospectively reviewed from our hospital’s database to identify cases of BMC and evaluate their association with TMJ and disc pathologies.

## 2. Materials and methods

This cross-sectional, observational, analytical study was conducted retrospectively. Patients who underwent brain MRI examinations, including the T2-weighted 3D SPACE sequence, at the Department of Radiology at Düzce University Hospital between 01 June 2023 and 01 June 2024 stemming from any clinical complaint were investigated.

Patients who underwent brain MRI examinations without the T2-weighted 3D SPACE sequence, as well as those with a history of trauma or fractures to the jawbone (either known or detected on MRI), previous TMJ surgery, or active rheumatic disease affecting the TMJ, were excluded from the study. As this was a retrospective study and as there are not many methodologically comparable large-scale studies on BMC available in the literature (most reports being case series), it was not possible to perform a prior power analysis or sample size calculation. Instead, all eligible patients within the study period were included in the analysis, resulting in a total of 29 BMC cases examined.

### 2.1 MRI technique

The MR imaging scans of all patients included the following sequences: axial turbo spin-echo (TSE) T1, axial TSE T2, coronal TSE T2, axial T2 fluid-attenuated inversion recovery (FLAIR) and diffusion-weighted imaging (DWI). Due to the long-standing integration of the sagittal T2-weighted 3D SPACE sequence into routine brain MRI examinations, this sequence was also performed for all subjects in this study. The parameters for the T2-weighted 3D SPACE sequence were as follows: time repetition/time to echo (TR/TE) = 3200/400 ms; variable flip angle; slice thickness = 1 mm; and field of view (FOV) = 256 × 256 mm.

### 2.2 Image evaluation

All brain MR examinations were performed by using a 3-Tesla MR system (Magnetom Skyra; Siemens Healthcare, Erlangen, BY, Germany). High-resolution monitors were employed to analyze all MR images of the brain stored in the hospital system. Each image was evaluated by a single neuroradiologist with over 15 years’ experience. Diagnoses were based solely on radiographic features via conventional MRI interpretation methods.

In cases diagnosed with BMC, the TMJ was carefully assessed. TMJ disorders showing pathological findings were classified into six categories for further analysis (see in Figs. 1,2).

**Fig. 1.** 3D CT and 3D T2-weighted SPACE MR images of a bifid mandibular condyle. Anterior (A) and posterior (B) projection 3D volume-rendered CT images demonstrating a left bifid mandibular condyle (arrows). Coronal (C) and sagittal (D and E) plane T2-weighted 3D SPACE images show a normal condyle on the right (single blue arrow) and a bifid mandibular condyle on the left (double blue arrows). On the right, the TMJ disc (double red arrows) appears normal in position and morphology, whereas on the left, the disc (single red arrow) is degenerated and deformed.

**Fig. 2.** T2-weighted 3D SPACE MR findings of the temporomandibular joint in a case of bifid mandibular condyle. Coronal (A) and sagittal (B and C) plane T2-weighted 3D SPACE images demonstrating bilateral bifid mandibular condyles (double blue arrows). The mandibular condyles are positioned in a subluxed appearance toward the level of the articular eminence. On the right, the TMJ disc is thickened, whereas on the left, the disc shows increased signal intensity (double red arrows).

MRI Diagnosis Categories:

1. Degeneration of the TMJ disc.

2. Displacement of the TMJ disc.

3. Deformation of the TMJ disc.

4. Degenerative osteoarthritis of the TMJ.

5. Effusion of the TMJ.

6. Subluxation of the TMJ.

### 2.3 Data analysis

A variety of statistical methods were employed to analyse the data. First, the descriptive statistics were applied and the numerical variables were reported as medians, minimums, maximums, means and standard deviations, while the categorical variables were reported as counts and percentages. Histogram plots were used to examine the distributions of numerical variables, and the Mann-Whitney U test was employed to evaluate these variables. Categorical variables were analyzed using the chi-squared test. A *p*-value of less than 0.05 was considered statistically significant. All analyses were performed by using SPSS version 23.0 (IBM Corp., Armonk, NY, USA).

## 3. Results

The study included 29 patients, of whom 9 (31%) were male and 20 (69%) were female. The median age was 58 years (range 19–76 years). Unilateral BMC was detected in 17 patients (58.6%) while 12 patients (41.4%) had bilateral BMC. Of the unilateral cases, BMC was observed on the right side of 10 patients (34.5%) and on the left side in 7 patients (24.1%).

No pathological findings were observed on MRI scans in 5 patients (17.2%). However, 24 patients (82.8%) exhibited at least 1 pathological MRI finding related to the TMJ or disc. 1 patient (3.4%) presented with all of the pathological findings evaluated in this study. Of the patients with pathological findings, 22 (75.9%) had TMJ disc degeneration; 8 (27.6%) had TMJ disc displacement; 10 (34.5%) had TMJ disc deformation; 2 (6.9%) had degenerative osteoarthritis of the TMJ; 12 (41.4%) had TMJ effusion; and 5 (17.2%) had TMJ subluxation.

The relationships between patient gender, the location of the BMC, and the presence of TMJ disc and joint disorders were analyzed in terms of their ages. Only patients with TMJ disc displacement exhibited significantly higher age groups (*p* = 0.008) (see Table 1). When the location of BMC and descriptive TMJ disc and joint disorders were analyzed according to patient gender, no statistically significant differences were observed (*p * > 0.05) (Table 2).

**Table 1.** Evaluation of gender, location of the bifid mandibular condyle, and descriptive disc and joint disorders according to patient age.

		Age			*p*
		Median	Min.	Max.	
Gender					
	Male	58.0	48.0	74.0	0.382
	Female	57.5	19.0	76.0	
Condylar side					
	Right	53.0	25.0	76.0	0.645
	Left	63.0	19.0	69.0	
	Bilateral	57.5	37.0	74.0	
Condylar position					
	Unilateral	59.0	19.0	76.0	0.739
	Bilateral	57.5	37.0	74.0	
Degeneration of the TMJ disc					
	None	57.0	25.0	70.0	0.646
	Present	58.5	19.0	76.0	
Displacement of the TMJ disc					
	None	56.0	19.0	70.0	**0.008**
	Present	66.0	54.0	76.0	
Deformation of the TMJ disc					
	None	59.0	25.0	74.0	0.982
	Present	55.0	19.0	76.0	
Degenerative osteoarthritis of the TMJ					
	None	58.0	19.0	76.0	0.829
	Present	59.5	54.0	65.0	
Effusion of the TMJ					
	None	63.0	25.0	74.0	0.080
	Present	54.0	19.0	76.0	
Subluxation of the TMJ					
	None	60.0	19.0	76.0	0.470
	Present	56.0	41.0	65.0	
Temporomandibular disorders					
	Present	57.5	19.0	76.0	0.817
	None	63.0	25.0	70.0	

*The Mann-Whitney test was used. TMJ: Temporomandibular joint; Min.: minimum; Max.: maximum. p-values shown in bold indicate statistically significant differences (p < 0.05).*

**Table 2.** Evaluation of the location of the bifid mandibular condyle and descriptive disc and joint disorders according to patient gender.

		Gender				
		Male		Female		*p*
		n	%	n	%	
Condylar side						
	Right	2	22.22	8	40.00	0.543
	Left	2	22.22	5	25.00	
	Bilateral	5	55.56	7	35.00	
Condylar position						
	Unilateral	4	44.44	13	65.00	0.298
	Bilateral	5	55.56	7	35.00	
Degeneration of the TMJ disc						
	None	3	33.33	4	20.00	0.438
	Present	6	66.67	16	80.00	
Displacement of the TMJ disc						
	None	6	66.67	15	75.00	0.642
	Present	3	33.33	5	25.00	
Deformation of the TMJ disc						
	None	8	88.89	11	55.00	0.076
	Present	1	11.11	9	45.00	
Degenerative osteoarthritis of the TMJ						
	None	9	100.00	18	90.00	0.326
	Present	0	0.00	2	10.00	
Effusion of the TMJ						
	None	5	55.56	12	60.00	0.822
	Present	4	44.44	8	40.00	
Subluxation of the TMJ						
	None	8	88.89	16	80.00	0.558
	Present	1	11.11	4	20.00	
Temporomandibular disorders						
	None	2	22.22	3	15.00	0.634
	Present	7	77.78	17	85.00	

*The Chi-square test was used. TMJ: Temporomandibular joint.*

Similarly, when BMC location and descriptive disc and joint disorders were examined in relation to the condylar position, no statistically significant differences were found (*p * > 0.05) (Table 3).

**Table 3.** Investigation of disc and joint disorders as defined by the location of the bifid mandibular condyle.

		Condylar position				
		Unilateral		Bilateral		*p*
		n	%	n	%	
Degeneration of the TMJ disc						
	None	4	23.53	3	25.00	0.927
	Present	13	76.47	9	75.00	
Displacement of the TMJ disc						
	None	13	76.47	8	66.67	0.561
	Present	4	23.53	4	33.33	
Deformation of the TMJ disc						
	None	10	58.82	9	75.00	0.367
	Present	7	41.18	3	25.00	
Degenerative osteoarthritis of the TMJ						
	None	16	94.12	11	91.67	0.798
	Present	1	5.88	1	8.33	
Effusion of the TMJ						
	None	10	58.82	7	58.33	0.979
	Present	7	41.18	5	41.67	
Subluxation of the TMJ						
	None	15	88.24	9	75.00	0.353
	Present	2	11.76	3	25.00	
Temporomandibular disorders						
	None	3	17.65	2	16.67	0.945
	Present	14	82.35	10	83.33	

*Chi-square test was used. TMJ: Temporomandibular joint.*

The statistical analysis of the relationships among descriptive disc and joint disorders is presented in Table 4. Notably, TMJ disc degeneration was significantly more common in BMC patients with TMJ disc deformation (*p* = 0.028).

**Table 4.** Evaluation of the relationship between the findings of descriptive disc and joint disorders in patients.

Evaluation of the relationships among temporomandibular joint and disc disorders in patients.						
Degeneration of the TMJ disc						
		None		Present		*p*
		n	%	n	%	
Displacement of the TMJ disc						
	None	7	100.00	14	63.64	0.061
	Present	0	0.00	8	36.36	
Deformation of the TMJ disc						
	None	7	100.00	12	54.55	**0.028**
	Present	0	0.00	10	45.45	
Degenerative osteoarthritis of the TMJ						
	None	7	100.00	20	90.91	0.408
	Present	0	0.00	2	9.09	
Effusion of the TMJ						
	None	5	71.43	12	54.55	0.430
	Present	2	28.57	10	45.45	
Subluxation of the TMJ						
	None	7	100.00	17	77.27	0.166
	Present	0	0.00	5	22.73	
Displacement of the TMJ disc						
		None		Present		*p*
		n	%	n	%	
Deformation of the TMJ disc						
	None	16	76.19	3	37.50	0.051
	Present	5	23.81	5	62.50	
Degenerative osteoarthritis of the TMJ						
	None	21	100.00	6	75.00	**0.018**
	Present	0	0.00	2	25.00	
Effusion of the TMJ						
	None	12	57.14	5	62.50	0.793
	Present	9	42.86	3	37.50	
Subluxation of the TMJ						
	None	18	85.71	6	75.00	0.495
	Present	3	14.29	2	25.00	
Deformation of the TMJ disc						
		None		Present		*p*
		n	%	n	%	
Degeneration of the TMJ disc						
	None	19	100.00	8	80.00	**0.043**
	Present	0	0.00	2	20.00	
Effusion of the TMJ						
	None	13	68.42	4	40.00	0.140
	Present	6	31.58	6	60.00	
Subluxation of the TMJ						
	None	17	89.47	7	70.00	0.187
	Present	2	10.53	3	30.00	
Degeneration of the TMJ disc						
		None		Present		*p*
		n	%	n	%	
Effusion of the TMJ						
	None	16	59.26	1	50.00	0.066
	Present	11	40.74	1	50.00	
Subluxation of the TMJ						
	None	24	88.89	0	0.00	**0.001**
	Present	3	11.11	2	100.00	
Effusion of the TMJ						
		None		Present		*p*
		n	%	n	%	
Subluxation of the TMJ						
	None	14	82.35	10	83.33	**0.005**
	Present	3	17.65	2	16.67	

*Chi-square test was used. TMJ: Temporomandibular joint. p-values shown in bold indicate statistically significant differences (p < 0.05).*

In BMC patients with TMJ disc displacement, the incidence of TMJ degenerative osteoarthritis was significantly higher (*p* = 0.018).

In BMC patients with TMJ disc deformation, the incidence of TMJ degenerative osteoarthritis was also significantly higher (*p* = 0.043).

In BMC patients with TMJ subluxation, the incidences of TMJ degenerative osteoarthritis and TMJ effusion were significantly higher (*p* = 0.001 and 0.005, respectively).

## 4. Discussion

3D imaging is the most reliable method for the morphological evaluation of BMC, in terms of allowing accurate differentiation of degenerative changes, cysts, tumors, metastatic lesions, or condylar fractures [23, 24, 25]. With the increasing use of multiplanar scanning and reconstruction techniques in CT and MRI, the detection and characterization of bifid mandibular condyles have become more common [1, 13, 26]. It became apparent with the study that utilization of the T2-weighted 3D SPACE sequence for the evaluation of BMC is primary. In the literature, MRI evaluations of BMC are typically reported as case studies [10, 13, 27, 28].

It is evaluated with the study that the TMJ in 29 identified BMC cases. Prior to 2005, a total of 45 BMC cases had been reported in the literature, and this number increased to 112 cases by 2011 [11, 24]. Nearly half of the prevalence studies on BMC have been conducted in Turkish populations [5]. Sahman *et al*. [29] suggested that BMC may be more common among the Turkish population. The study was conducted among Turkish population, but because studies from other countries are limited, no conclusions can be drawn regarding potential racial or ethnic differences represented in BMC [5].

Bilateral BMCs are even rarer [11]. Studies reporting on unilateral and bilateral occurrences indicate a ratio of approximately 3:1, whereas cadaver studies suggest a ratio of 4.6:1 [11, 30, 31]. Studies conducted in Turkish populations have shown that the number of unilateral cases is 5.4–13.1 times higher than the number of bilateral cases [24, 32]. By contrast, the study found that the rate of unilateral occurrence was 58.6%, while the rate of bilateral occurrence was 41.4%. This higher rate may be related to the developmental characteristics of BMC, the exclusion of patients with a history of trauma, and the use of the 3D SPACE technique, which allows a more detailed evaluation via thin-section imaging.

Notably, the relationship between BMC and gender is inconsistent throughout the literature. Some studies report that BMC is 1.5–3.5 times more prevalent in women than in men [23, 33, 34], whereas in the study, the ratio was approximately 2:1. Conversely, other studies have reported similar rates for both gender [19, 29, 31, 32]. However, Antoniades *et al*. [35] reported that BMC was approximately 1.5 times more prevalent in men. These inconsistencies may be due to factors such as small sample sizes resulting from the rarity of BMC, the use of different imaging techniques and potential racial differences.

Some studies [18, 19] have reported a higher prevalence of unilateral BMC on the left side, while others [29, 31, 36] have reported a higher prevalence on the right side. In this study, 34.5% of patients had right-sided BMC and 24.1% had left-sided BMC, indicating a higher prevalence on the right side. However, this difference was not statistically significant (*p * > 0.05), aligning with the findings of other related studies in the literature [18, 29].

In a study conducted by Rehman *et al*. [37], 37 cases of TMJ ankylosis were identified through the use of CT scans. BMC was present in 10 of these cases. In the present study, however, no cases of TMJ ankylosis were observed among patients with BMC. This may be because we excluded patients with a history of trauma, whereas most cases of BMC associated with TMJ ankylosis reported in the literature involved an underlying traumatic event [11, 15, 37].

A case report published in 2021 found that a 38-year-old male patient with left-sided BMC had mild degenerative changes in the TMJ disc, as well as small cystic findings. Following the manifestation of symptoms, a control MRI scan revealed impingement in the anterior or intermediate disc band, as well as minimal anterior translation, both of which were caused by the left BMC. Based on these findings, the authors hypothesized that the bifid condyle may also play a role in disc degeneration, in addition to age-related factors. They also concluded that a relationship between BMC and temporomandibular disorders needs to be established [10].

A systematic review and meta-analysis published in 2023 reported that the correlation between BMC and TMJ pathology was relatively low in studies with large sample sizes [1]. In contrast, in the present study, 82.8% of BMC patients exhibited at least one pathological MRI finding associated with the TMJ or disc. In most studies included in the meta-analysis, BMC cases were identified and evaluated using panoramic radiography, of which may have led to misinterpretation. Furthermore, only three MRI-based studies were included, with 42 cases in total [1].

In a study conducted by Cho *et al*. [18], which examined 44 cases of BMC using CT, no statistically significant difference was found by the distribution of clinical symptoms between normal-shaped condyles and BMC cases, suggesting the idea that BMC does not cause TMJ symptoms. The only statistically significant difference between normal and BMC condyles was observed in the number of osteophytes in symptomatic patients, with normal condyles exhibiting more osteophytes than BMC condyles. Based on these findings, it is hypothesized that the groove in BMC tends to flatten the condyle, which may reduce its potential for osteophyte formation [18]. In the present study, the rarest pathological finding among BMC patients was TMJ degenerative osteoarthritis, which was observed in only two patients, consistent with the findings of Cho *et al*. [18]. Notably, Cho *et al*. [18] used CT for TMJ evaluation; however, MRI is considered a more precious method for assessing TMJ and disc pathology [29, 38]. In our study, disc degeneration was observed in approximately three-quarters of BMC patients. Furthermore, disc degeneration was a more common feature among patients with disc deformities (*p* = 0.028), suggesting a potential association between disc degeneration and structural deformity.

In a case report of bilateral BMC conducted by Alpaslan *et al*. [28], MRI findings revealed bilateral anterior disc displacement without reduction. In our study, 8 patients (27.6%) exhibited disc displacement.

Most BMC cases are asymptomatic and are detected during routine dental radiographic examinations [5, 6, 37]. However, some cases involving patients presenting with TMJ symptoms, swelling, trauma, or ankylosis have also been reported [6, 10, 11, 23].

One major limitation of this study is the lack of assessment of clinical symptoms of temporomandibular disorders (*e.g.*, pain or functional limitation) and the absence of detailed physical examinations, which restricts the ability to determine whether the MRI findings represent clinically significant pathology. Although most BMC cases demonstrated MRI abnormalities, these changes should be interpreted with caution, as disc degeneration or effusion can also be observed in asymptomatic individuals. Thus, imaging findings alone are not sufficient for the diagnosis of temporomandibular disorders (TMD). Nevertheless, BMC may alter condylar morphology and joint biomechanics, potentially leading to functional problems with time. In addition, the study employed a descriptive retrospective design and did not include a control group, which prevents concluding remarks on whether TMJ abnormalities are more frequent in BMC patients compared with the general population or not. Finally, TMJ evaluation was based on brain MRI examinations rather than dedicated oblique sagittal or coronal TMJ sequences, which may have reduced the accuracy of assessing subtle anatomical relationships. Future prospective multicenter studies integrating both imaging and clinical evaluations are suggested to provide a more comprehensive understanding of the relationship between BMC and TMJ disorders.

## 5. Conclusions

In the majority of BMC cases, at least one pathological MRI finding related to the TMJ or disc is observed, with TMJ disc pathology being the most common. In this context, BMC may contribute to the etiology of temporomandibular disorders or exacerbate existing conditions. However, further studies with larger sample sizes are suggested to confirm these findings.
